# The complete recanalization of PICC-related venous thrombosis in cancer patients: A series of case reports

**DOI:** 10.3892/etm.2013.1150

**Published:** 2013-06-07

**Authors:** ZUO-PING HUANG, XING-JING LIU, BIN-XIN ZOU, LI-GEN WANG, TAO ZHOU

**Affiliations:** Department of Oncology, The Affiliated Wujing Hospital of Guangzhou Medical College, State Key Laboratory of Respiratory Disease, Guangzhou, Guangdong 510507, P.R. China

**Keywords:** peripherally inserted central catheter, venous thrombosis, *Panax notoginseng* saponins

## Abstract

In this study, cancer patients with venous thrombosis associated with the use of peripherally inserted central catheters (PICCs) underwent complete recanalization by the administration of *Panax notoginseng* saponins (PNS), which vary from heparin or urokinase in that they do not have the same risks associated with thrombolysis, including bleeding. To the best of our knowledge, this is the first study concerning the treatment of cancers with PNS to be reported in the literature. Three cancer patients aged 30–50 years old, two females and one male, were subjected to chemotherapy. On the first day of chemotherapy, a PICC was inserted into the right basilic vein with its tip in the superior vena cava. On the third day of chemotherapy, pain, swelling and skin flushing started. In the following days, particularly days 10–13, a Doppler ultrasound examination confirmed a long thrombus along the PICC line in the axillary vein and brachial veins in each patient. The patients rejected the insertion of an inferior vena cava filter, and neither heparin nor urokinase were administered due to contra-indications. An injection of PNS (200 mg) was administered every day. On days 20–28 of chemotherapy, the thrombus in the axillary and brachial veins disappeared in the three patients. It was concluded that PNS promote blood circulation, which prevents blood stasis and reduces the toxicity of cisplatin. The results suggest that PNS are a feasible and effective treatment option for many types of cancer, but have a broader clinical impact on cancer patients with PICC-related venous thrombosis. Therefore, this study is an original case report of particular interest to cancer patients with PICC-related venous thrombosis.

## Introduction

Superficial veins are often damaged by chemotherapy in patients with malignant tumors. Peripherally inserted central catheters (PICCs) are favored clinically as they cause minimal vascular damage, particularly in the chemotherapy of patients with malignant tumors (1). However, certain patients experience complications, including deep vein thrombosis, infection and pulmonary embolisms. Therefore, a number of management strategies have been recommended in order to prevent and treat these complications (2). Heparin and urokinase have been widely used to prevent these side-effects (3); however, certain patients may not be treated with heparin or urokinase due to contraindications.

To the best of our knowledge, no studies are currently available regarding the contraindications of heparin or urokinase in PICC-related venous thrombosis in cancer patients. In the present study, the use of *Panax notoginseng* saponins (PNS) to treat PICC-related venous thrombosis in cancer patients was investigated.

## Case reports

A 50-year-old female with cervical cancer, a 31-year-old female with brain metastases and a 47-year-old male with lung cancer were admitted for chemotherapy. All patients were Cantonese. The cervical cancer patient had irregular vaginal bleeding and the lung cancer patient had hemoptysis. Written informed consent was obtained from all patients. This study is approval by by the ethics committee of the Affiliated Wujing Hospital of Guangzhou Medical College.

**Figure 1.**
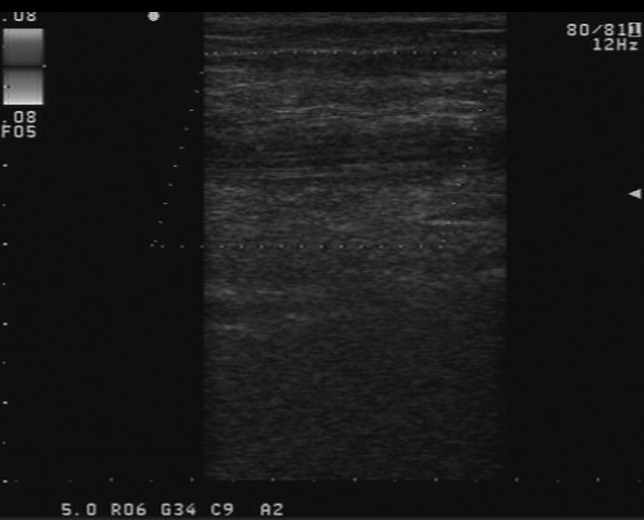
Color doppler ultrasound displays venous thrombosis in the brachial vein.

**Figure 2.**
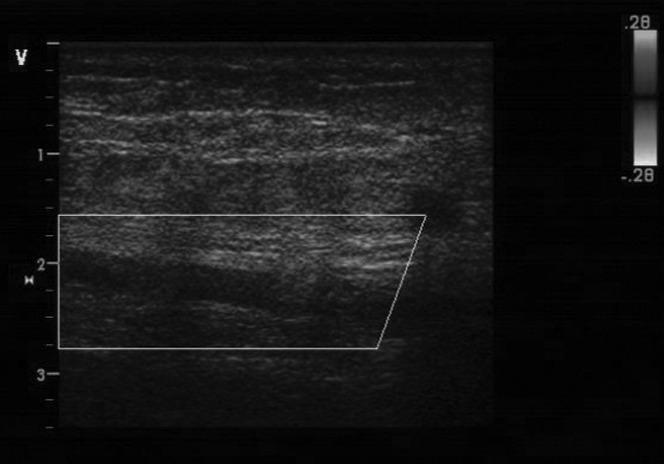
Color doppler ultrasound reveals that the brachial vein static wall is smooth and the thrombus has disappeared. Same patient as shown in Fig 1.

On the first day of chemotherapy, the hemoglobin levels of the patients were in the range of 101 to 142 g/l, while the white blood cell counts were 4.8–7.8×10^9^ cells/l and the platelet counts were 102.5–204×10^9^/l. A 4-F double lumen PICC was inserted into the right basilic vein on the first day of chemo-therapy with its tip in the superior vena cava. On the third day of chemotherapy, pain, swelling and skin flushing began in the upper right extremity at the puncture site in all three patients. The following day, one patient had a fever of 38.5˚C and aspirin was administered. On days 10–13 of chemotherapy, Doppler ultrasound examination confirmed a long thrombus along the PICC line in the axillary and brachial veins in the three patients (Fig. 1). The patients rejected the insertion of an inferior vena cava filter. The patients were administered a recombinant tissue plasminogen activator, and a mean platelet count of 28×10^9^/l and contraindications for heparin or urokinase were observed. A complete blood count revealed that the hemoglobin level was 118–132 g/l and the white blood cell count was 8.02–12.8×10^9^ cells/l. The patients were treated with an injection of ceftazidime 1.0 g every 12 h, 50 mg asprin administered orally and a 200 mg injection of PNS every day. On days 20, 27 and 28 of chemotherapy, the thrombus in the axillary and brachial veins disappeared in a 50-year-old female with cervical cancer, a 31-year-old female with brain metastases and a 47-year-old male with lung cancer, respectively (Fig. 2). The cost of the PNS treatment was ¥1290.3, which is lower than that of urokinase (¥2581.2).

## Discussion

PICCs are favored clinically as they cause minimal vascular damage, particularly in the chemotherapeutic treatment of malignant tumors (1). However, in certain patients, complications, including deep vein thrombosis, infection and pulmonary embolisms occur. Therefore, numerous management strategies have been recommended in order to prevent and treat these complications. Previous studies have demonstrated that the time, size and repeated intubation of PICCs increase the risk of venous thrombosis (4–6). We identified that pain, swelling and skin irritation were key factors in the formation of vein thrombosis.

We have considered three options for the management of patients with PICC-related thrombosis: surgery, interventional radiology-guided clot retrieval and thrombolysis. A surgical consultant deemed that the patients were not suitable candidates for surgery, as they had coronary heart disease and diabetes. The three patients did not wish to undergo interventional radiology to investigate the possibility of removing the clot. Consequently, we considered systemic thrombolysis as a treatment strategy for the patients. The patients were not suitable for treatment with heparin or urokinase due to contraindications, including irregular vaginal bleeding and hemoptysis. Therefore, heparin and urokinase were not used due to the risks associated with bleeding, hematoma and intracranial hemorrhage. Treatment with PNS was selected, as PNS promote blood circulation, which prevents blood stasis and may clean and activate the channels and collaterals (7). A previous study has demonstrated that PNS enhance the cytotoxicity of cisplatin by increasing the gap junction and intercellular communication (8). The PNS treatment is low in cost and easy to administer. PNS have been widely used in the treatment of patients with cerebral and myocardial infarction in China. On average by day 25 of chemotherapy, complete recanalization of the vein was observed.

In conclusion, our results suggest that PNS are a feasible and effective treatment option for various types of cancer, but have a broader clinical impact on cancer patients with PICC-related venous thrombosis. Therefore, this study is an original case report of interest to cancer patients with PICC-related venous thrombosis.
